# Entry of Herpes Simplex Virus Type 1 (HSV-1) into the Distal Axons of Trigeminal Neurons Favors the Onset of Nonproductive, Silent Infection

**DOI:** 10.1371/journal.ppat.1002679

**Published:** 2012-05-10

**Authors:** Wali Hafezi, Eva U. Lorentzen, Bodo R. Eing, Marcus Müller, Nicholas J. C. King, Barbara Klupp, Thomas C. Mettenleiter, Joachim E. Kühn

**Affiliations:** 1 University Hospital Münster, Institute of Medical Microbiology - Clinical Virology, Münster, Germany; 2 Interdisciplinary Center of Clinical Research (IZKF), Münster, Germany; 3 University Hospital Bonn, Department of Neurology, Bonn, Germany; 4 University of Sydney, Sydney Medical School, Department of Pathology, Bosch Institute for Medical Research, New South Wales, Australia; 5 Friedrich-Loeffler-Institut, Institute of Molecular Biology, Greifswald-Insel Riems, Germany; McMaster University, Canada

## Abstract

Following productive, lytic infection in epithelia, herpes simplex virus type 1 (HSV-1) establishes a lifelong latent infection in sensory neurons that is interrupted by episodes of reactivation. In order to better understand what triggers this lytic/latent decision in neurons, we set up an organotypic model based on chicken embryonic trigeminal ganglia explants (TGEs) in a double chamber system. Adding HSV-1 to the ganglion compartment (GC) resulted in a productive infection in the explants. By contrast, selective application of the virus to distal axons led to a largely nonproductive infection that was characterized by the poor expression of lytic genes and the presence of high levels of the 2.0-kb major latency-associated transcript (LAT) RNA. Treatment of the explants with the immediate-early (IE) gene transcriptional inducer hexamethylene bisacetamide, and simultaneous co-infection of the GC with HSV-1, herpes simplex virus type 2 (HSV-2) or pseudorabies virus (PrV) helper virus significantly enhanced the ability of HSV-1 to productively infect sensory neurons upon axonal entry. Helper-virus-induced transactivation of HSV-1 IE gene expression in axonally-infected TGEs in the absence of *de novo* protein synthesis was dependent on the presence of functional tegument protein VP16 in HSV-1 helper virus particles. After the establishment of a LAT-positive silent infection in TGEs, HSV-1 was refractory to transactivation by superinfection of the GC with HSV-1 but not with HSV-2 and PrV helper virus. In conclusion, the site of entry appears to be a critical determinant in the lytic/latent decision in sensory neurons. HSV-1 entry into distal axons results in an insufficient transactivation of IE gene expression and favors the establishment of a nonproductive, silent infection in trigeminal neurons.

## Author Summary

Upon primary infection of the oronasal mucosa, herpes simplex virus type 1 (HSV-1) rapidly reaches the ganglia of the peripheral nervous system via axonal transport and establishes lifelong latency in surviving neurons. Central to the establishment of latency is the ability of HSV-1 to reliably switch from productive, lytic spread in epithelia to nonproductive, latent infection in sensory neurons. It is not fully understood what specifically disposes incoming particles of a highly cytopathogenic, fast-replicating alphaherpesvirus to nonproductive, latent infection in sensory neurons. The present study shows that selective entry of HSV-1 into the distal axons of trigeminal neurons strongly favors the establishment of a nonproductive, latent infection, whereas nonselective infection of neurons still enables HSV-1 to induce lytic gene expression. Our data support a model of latency establishment in which the site of entry is an important determinant of the lytic/latent decision in the infected neuron. Productive infection of the neuron ensues if particles enter the soma of the neuron directly. In contrast, previous retrograde axonal transport of incoming viral particles creates a distinct scenario that abrogates VP16-dependent transactivation of immediate-early gene expression and precludes the expression of lytic genes to an extent sufficient to prevent the initiation of massive productive infection of trigeminal neurons.

## Introduction

Herpes simplex virus type 1 (HSV-1) and 2 (HSV-2) are prototypic members of the genus *Simplexvirus* within the herpesvirus subfamily *Alphaherpesvirinae*. *In vitro*, HSVs are pantropic, causing lytic infections in various tissues and cell types of a broad range of host species [1]. *In vivo*, humans are the only natural hosts of HSVs, and infection is almost exclusively limited to the epithelial cells and neurons of the peripheral nervous system (PNS). The portal of entry in HSV-1 infections is the oronasal mucosa, where the virus spreads rapidly with productive, lytic infection of epithelial cells [2]. HSV-1 reaches the PNS by entry into free nerve endings that are in contact with the infected epithelium, and by retrograde axonal transport [3]. Beyond the neonatal period, replication of HSV-1 within the PNS is tightly controlled, and further ascending spread into the central nervous system is prevented in the immunocompetent host. As a result, HSV-1 establishes latency in surviving neurons, converting them into a lifelong reservoir of recurrent infection, which occurs in response to diverse stimuli causing neuronal stress [4]. The ability to switch from rapidly progressing, lytic spread in epithelia to a nonproductive, latent infection in sensory neurons is fundamental to the life cycle of HSVs and other related alphaherpesviruses. Although crucial for our understanding of the pathogenesis of alphaherpesvirus infection, the events that ultimately trigger the establishment of latent infection in sensory neurons are not fully understood. The latency-associated transcript (LAT) is abundantly expressed in infected neurons, and has been shown to promote the establishment and maintenance of latency, in part because of the anti-apoptosis functions of LAT and the ability of micro-RNAs and other small non-coding RNAs encoded by LAT to interfere with productive infection [4].

The viral proteins ICP0 and VP16 have both been implicated in the lytic/latent decision in the infected neuron [5]. The HSV-1 immediate-early (IE) regulatory protein ICP0 is a RING finger E3 ubiquitin ligase that acts as a promiscuous activator of gene expression and is critical for the efficient initiation of productive infection, the prevention of cellular silencing of viral transcription, and reactivation from latency [6]. Central to the function of HSV-1 ICP0 and functional homologues in other alphaherpesviruses is the ability of ICP0 to degrade PML nuclear bodies (also known as nuclear domain 10, ND10) and to disrupt the ND10-dependent antiviral interferon response and other cellular functions linked to ND10 [7], [8]. HSV-1 ICP0 has been found to functionally interact with class II histone deacetylases (HDAC), to dissociate HDAC from the lysine-specific demethylase 1/REST/CoREST repressor complex, and to promote histone removal and acetylation, thus preventing the formation of inactive chromatin on the HSV-1 genome [9]–[11]. Posttranslational histone modifications have been implied in HSV gene expression during lytic and latent infection, and the establishment, maintenance, and reactivation from latency [12]–[16].

HSV-1 VP16 (also designated pUL48 or alpha-transinducing factor, αTIF) is a structural component of the tegument layer. Upon fusion with the cytoplasmic membrane of monolayer cells, HSV-1 particles deliver 500–1,000 molecules of VP16 into the cytoplasm, which are transported to the nucleus independently of capsids [17].

Although purified *in vitro* de-enveloped HSV-1 particles containing a VP16-EGFP fusion protein were reported to move in a retrograde direction along microtubules when injected into squid giant axons [18], several studies of HSV-1 and other alphaherpesviruses have demonstrated that VP16 dissociates from viral particles upon entry into the host cell and that capsids are transported to the nucleus independently of VP16 [19]–[21]. Live-cell imaging experiments examining the retrograde axonal transport of pseudorabies virus (PrV) and HSV-1 in neurons of human, mouse and avian origin have shown that VP16 and other proteins of the outer tegument layer are predominantly lost from the nucleocapsid prior to the onset of retrograde axonal transport, and do not move with the capsid to the nucleus [22].

However, it was also noted that to some extent VP16 appears to be axonally transported in retrograde direction independent of capsids.

In lytic infection, VP16 forms a tripartite complex with the cellular proteins HCF-1 and Oct-1, which binds to the TAATGARAT elements present in HSV IE promoters and acts as a potent transcriptional activator of IE gene expression [23]–[26]. The transcriptional activation domain of HSV-1 VP16 (VP16AD) interacts with a large number of cellular factors that are involved in gene activation [27]. Although not essential for IE gene expression, coactivators recruited by the HSV-1 VP16AD contribute to relatively low levels of histones on the viral genome during lytic infection [28]–[31]. VP16 is essential for stress-induced HSV-1 reactivation *in vitro* [32]. Exit from latency following heat shock in the mouse ocular model has been reported to depend on the *de novo* activation of the VP16 promoter and synthesis of VP16 in infected neurons [33]. In stressed neurons, HCF-1 has been shown to relocalize from the cytoplasm to the nucleus and to be recruited to HSV-1 IE promoters [34]. The regulated relocalization of *de novo* synthesized VP16 and HCF-1 from the cytoplasm to the nucleus of stressed neurons appears to be a critical step in the initiation of lytic gene expression during reactivation from latency [35]. In addition to its regulatory function in IE gene expression, VP16 and homologous alphaherpesvirus proteins of the outer tegument layer mediate essential functions related to viral egress [36].

At present, animal models allow only a pinpoint, snapshot-like observation of the critical early phase of viral arrival in the PNS and onset of replication. Furthermore, there is enormous variation in the outcome of HSV-1 infection of the nervous system in laboratory animals. In mice, the course of infection depends on various factors, including the viral strain, infectious dose, route of infection, mouse strain and age, and prior immunization history of the animal [37]. As an alternative to animal models, classical or modified Campenot chambers and microfluidic devices have been used to study the directional spread of alphaherpesviruses between epithelial cells and neurons in compartmentalized organotypic culture systems [38]–[42]. The directional spread of HSV-1 between epithelia and the PNS has been analyzed in our laboratory by establishing compartment cultures based on chick embryonic corneal epithelial cells and trigeminal ganglion (TG) explants (TGEs) [43].

The aim of the present study was to identify factors triggering either productive or silent infection of sensory neurons by mimicking the critical early phase of primary PNS infection in organotypic cultures *ex vivo*. Interestingly, remarkable differences in the ability of HSV-1 to initiate productive infection in sensory neurons were observed depending on the site used to infect the explants. In accordance with earlier reports [22], [43], [44], productive infection was easily induced in embryonic chicken neurons by directly adding the viral inoculum to the explant cultures. In contrast, upon entry into distal axons, incoming HSV-1 genomes were largely destined to a quiescent, latency-like infection, characterized by the expression of high levels of the latency-associated transcript (LAT). HSV-1 remained responsive to the IE gene transcriptional inducer hexamethylene bisacetamide (HMBA), and to transcriptional transactivation by simultaneous co-infection of the ganglion compartment (GC) with helper virus. Efficient transactivation was dependent on the presence of functional VP16 in HSV-1 helper virus particles. To our knowledge, this is the first report to provide experimental evidence that axonal infection and preceding retrograde axonal transport of subviral particles strongly diminishes the ability of HSV-1 to initiate a productive infection of neurons, most likely via a failure of VP16-induced expression of lytic genes.

## Results

### Experimental setup of the organ model explant cultures

**Figure 1:**
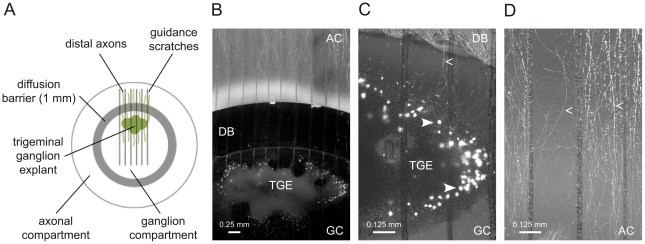
Trigeminal ganglion explant cultures. (A) Scheme of the double chamber system. (B–D) Trigeminal ganglion explant (TGE) cultures stained with DiI in the axonal compartment (AC) 48 h before imaging: (B) overview and (C, D) typical DiI-positive neurons (solid arrowheads) and neurites (arrowheads) in the ganglion compartment (GC) and AC shown at higher magnification. DB, diffusion barrier.

In the present study we analyzed the course of a synchronous HSV-1 infection of TGE cultures resulting from the application of a defined viral inoculum to either free nerve endings or directly to the explants. TGEs were derived from day-15 chicken embryos, as described previously [43]. Selective HSV-1 infection of distal axons was achieved with the aid of a Campenot-like compartment chamber [38], in which the inner compartment containing the TGE (i.e., the GC) was separated by a leak-proof diffusion barrier from the outer compartment containing the distal axons [the axonal compartment (AC)] (Figure 1A). Infection of the respective compartment was performed after 5–6 days of *in vitro* cultivation. Staining of the AC with the retrograde neuronal tracer DiI showed that at this time point approximately 200 neurites/TGE had crossed the diffusion barrier and reached the AC. Neurites within the AC were essentially free of accompanying glial cells. By 48 h after applying DiI to the AC, approximately 100 neurons/TGE could be visualized in intact cultures. DiI-positive neurons exhibited a characteristic morphology with a large, rounded soma, and were mostly localized at the margin of the explant. Many neurons contained prominently stained neurites (Figure 1B–D).

**Figure 2:**
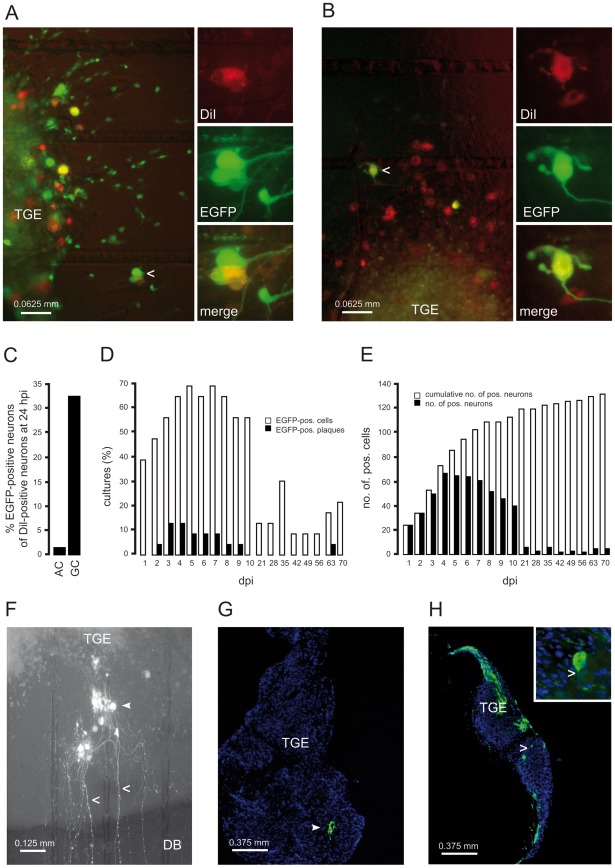
Protein expression and spread of HSV-1 in infected TGEs. (A) Infection of the GC with HSV-1 17 CMV-IEproEGFP. TGEs were infected with 1×10^6^ pfu, and stained in the AC with DiI at 1 hpi. At 24 hpi, DiI-specific (A, top right) and EGFP-specific (A, middle right) fluorescence was monitored in intact cultures. The arrowhead indicates a typical EGFP-expressing, DiI-positive neuron depicted at higher magnification at the right margin. A merged image of DiI- and EGFP-specific fluorescence is also shown (A, bottom right). (B) Infection of the AC with HSV-1 17 CMV-IEproEGFP. EGFP expression in single neurons at 48 hpi with HSV-1 17 CMV-IEproEGFP. TGEs were infected in the AC with 5×10^6^ pfu and stained with DiI at 1 hpi. A typical EGFP- and DiI-positive neuron is indicated by an arrowhead and depicted at higher magnification in the right-hand image. (C) Percentages of DiI/EGFP-double positive neurons. TGEs were either infected in the GC or in the AC with HSV-1 17 CMV-IEproEGFP and co-stained with DiI as given in (A,B). At 24 hpi, the numbers of EGFP and DiI-positive neurons in intact TGEs were determined microscopically. (D) Percentages of TGEs containing EGFP-positive (pos.) cells (white bars) and plaque-like clusters of EGFP-positive cells (EGFP-pos. plaques) (black bars). TGEs (*n* = 23) were axonally-infected with 5×10^6^ pfu of HSV-1 17 CMV-IEproEGFP and monitored daily from 1 to 10 dpi, and from 3 to 10 wpi. (E) Time kinetics of EGFP expression in neurons. Reporter gene expression in the TGEs shown in (D) was documented photographically at the time points indicated. Individual EGFP-positive neurons were identified, and the total (black bars) and cumulative (white bars) numbers of EGFP-positive neurons were determined. (F) Plaque-like cluster of EGFP-positive cells in an axonally-infected TGE at 5 dpi. EGFP-positive cells (triangle) and typical, EGFP-positive neurites (arrow heads) are indicated. (G) Detection of HSV-1-positive cells in cryosections of infected TGEs. TGEs were infected in the AC with 5×10^6^ pfu of HSV-1 17syn^+^. HSV-1-positive cells were detected at 2 dpi by immunofluorescence using a polyclonal antibody against HSV-1 (green), nuclei were stained with 4′,6-diamidino-2-phenylindole (blue). (H) TGEs were infected in the GC with 10^4^ pfu of HSV-1 17syn^+^, and cultures were fixed and stained at 2 dpi, as given above. A typical HSV-1-positive neuron is shown at higher magnification in the inset image; the neurite is indicated by the arrowhead.

Direct infection of the GC with HSV-1 17 CMV-IEproEGFP expressing EGFP under control of the CMV IE-promoter, and monitoring of the reporter protein expression in intact cultures by fluorescence microscopy demonstrated that embryonic chicken TGEs are susceptible to productive HSV-1 infection under the culture conditions used. By 24 h after infection [hours post infection (hpi)] of the GC with 1×10^6^ plaque-forming units (pfu) of HSV-1 17 CMV-IEproEGFP, the vast majority of cells directly accessible to the virus inoculum strongly expressed EGFP. Costaining of the AC with DiI allowed the identification of EGFP-positive neurons with neurites reaching the AC (Figure 2A). Approximately 30% of DiI-positive neurons (53 out of 163 neurons) were found to be EGFP-positive at 24 hpi (Figure 2C). According to their morphology, most of the remaining DiI-negative, EGFP-positive cells appeared to be macroglial cells, most likely Schwann cells.

### Selective infection of the AC

We next studied the retrograde spread of HSV-1 in intact TGEs by the addition of HSV-1 17 CMV-IEproEGFP to the AC and daily monitoring of cultures by fluorescence microscopy.

In preliminary experiments, various infectious doses and protocols for infection of the AC were evaluated. Except where indicated otherwise, an inoculum of 5×10^6^ pfu of HSV-1 added for 1 h to the AC was used in all subsequent experiments. This infectious dose corresponded to a multiplicity of infection (MOI) of approximately 5 in a monolayer culture in a 35-mm-diameter dish, and did not exhibit unwanted stimulatory effects on the TGEs due to serum components present in the virus stocks. Replacement of the TG medium in the AC by serum-containing media stimulated the outgrowth of glial cells or fibroblasts into the AC and occasionally led to the contraction and partial detachment of the explants (data not shown).

As shown in Figure 2B, the diffusion barrier proved to be tight, effectively inhibiting the nonspecific infection of cells that surrounded the explant, and were in direct contact with the inner rim of the glass cylinder. However, EGFP expression in the TGEs was unexpectedly poor. At 24 hpi, less than half of the axonally-infected cultures contained single EGFP-positive cells. Staining of the AC with DiI immediately after infection showed that EGFP expression was restricted to just a few isolated neurons (Figure 2B). Less than 2% of DiI-positive neurons (2 out of 127 neurons) were found to be EGFP-positive (Figure 2C).

The low number of reporter protein expressing neurons in cultures axonally-infected with HSV-1 17 CMV-IEproEGFP at 24 hpi prompted us to monitor the kinetics of EGFP expression in intact TGEs over a longer time period (Figure 2D–F). During the first 10 days after infection [days post infection (dpi)], the percentage of cultures containing EGFP-positive cells slowly increased from approximately 40% on 1 dpi to approximately 70% between 4 and 6 dpi, and decreased thereafter. Between 2 and 8 dpi, small plaque-like clusters of EGFP-positive cells appeared in approximately 15% of cultures, persisted for a few days, and disappeared thereafter, indicating a self-limiting secondary spread of HSV-1 within the TGEs (Figure 2D, F). In axonally infected TGEs, cell free virus could not be isolated from the supernatants of the GC (data not shown).

The long-term monitoring of these cultures at weekly intervals showed that in 10–20% of cultures, low and fluctuating levels of EGFP-expressing neurons persisted up to 10 weeks postinfection (wpi); in one culture a plaque-like cluster of infected cells developed at 9 wpi (Figure 2D). The onset of EGFP expression in individual axonally-infected neurons was followed up with the aid of serial photographic documentation. This demonstrated that the number of EGFP-expressing neurons peaked at 4 dpi, and then decreased to low and fairly stable levels thereafter (Figure 2E). Determination of the cumulative number of EGFP-positive neurons revealed differences in the kinetics of the onset of EGFP expression, with an approximately fivefold increase in the cumulative number of positive neurons between 1 and 7 dpi, and a long-lasting but minimal (i.e., less than 1.1-fold) increase between 3 and 10 wpi (Figure 2E).

As demonstrated in Figure 2G and H, the spread of the parental wild-type (wt) strain HSV-1 17syn^+^ closely resembled the pattern of fluorescence observed in HSV-1 17 CMV-IEproEGFP-infected TGEs. Two days after infection of the AC, single cells or small plaque-like clusters of HSV-1-infected cells located in the interior of explants could be detected in a few TGEs (Figure 2G). In contrast, after direct infection of the GC with 10^4^ pfu of HSV-1 17syn^+^, spread of HSV-1 into the interior parts of TGEs was observed, and large areas of HSV-1-positive cells containing infected glial cells and neurons could be visualized (Figure 2H).

### Viral genome and transcript levels in infected TGEs

**Figure 3:**
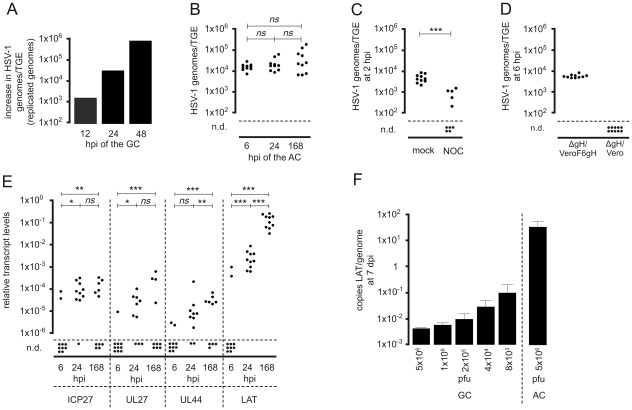
Genome and transcript levels in HSV-1-infected TGEs. (A) Replication of HSV-1 17syn^+^ in TGEs infected in the GC. Groups of three TGEs were infected in the GC with 1×10^4^ pfu of HSV-1 17syn^+^. TGEs were harvested at the time points indicated. DNA extracts were pooled, genome levels per TGE were quantified by qPCR, and the increase in genome levels relative to that at 1 hpi was determined (replicated genomes). (B) Genome levels in TGEs infected in the AC with HSV-1 17syn^+^. Groups of ten cultures, each containing two TGEs, were infected with 5×10^6^ pfu of HSV-1 17syn^+^, TGEs of individual cultures were pooled and genome levels/TGE were determined by qPCR at the time points indicated, n.d., No viral genomes detected by qPCR. The statistical significances of results are indicated as follows: *ns* (not significant), *P*>0.05; *P = 0.005–0.05; **P = 0.0005–0.005; ***P<0.0005. The median genome levels did not differ significantly between 6 hpi and 24 hpi, 24 hpi and 7 dpi, and 6 hpi and 7 dpi (*P* = 0.393, 0.4813, and 0.2176, respectively; Mann-Whitney test). (C) Effect of nocodazole on the axonal transport of viral particles. Cultures were infected in the AC with 5×10^6^ pfu of HSV-1 17syn^+^ in the presence of 10 µM nocodazole (NOC); mock-treated cultures served as controls. TGEs were harvested at 2 hpi, and genome levels were determined by qPCR, n.d., No viral genomes detected by qPCR. Differences in genome levels between NOC-treated and untreated cultures at 2 hpi were highly significant (*P* = 0.0002; unpaired *t* test with Welch correction). (D) Infection of the AC with the fusion-deficient, gH-negative mutant HSV-1 KOSgH87. Infection of the AC was performed with 1×10^8^ particles of HSV-1 KOSgH87. The virus was purified using a saccharose gradient from the supernatants of transcomplementing VeroF6gH cells (ΔgH/VeroF6gH) (corresponding to 5×10^6^ pfu on VeroF6gH cells) and of noncomplementing Vero cells (ΔgH/Vero) (corresponding to 10 pfu on VeroF6gH cells). Genome levels were determined by qPCR at 6 hpi. (E) Transcript levels in TGEs axonally-infected with HSV-1 17syn^+^. Groups of ten cultures, each containing two TGEs, were infected in the AC with 5×10^6^ pfu. IE (ICP27), E/L (UL27), L (UL44), and LAT transcripts were quantified by RT-PCR and normalized to β-actin transcript levels. Transcript levels at 6, 24, and 168 hpi (7 dpi) are shown, n.d., No viral transcripts detected. The statistical significances of differences in median transcript levels between 6 and 24 hpi, 24 and 168 hpi, and 6 and 168 hpi are indicated (ICP27: *P* = 0.0068, 0.8798, and 0.0011, respectively; UL27: *P* = 0.0433, 0.1124, and 0.0001, respectively; UL44: *P* = 0.1230, 0.0021, and <0.0001, respectively; LAT: *P*<0.0001, <0.0001, and <0.0001, respectively; Mann-Whitney test). (F) Specific transcriptional activity of the LAT gene in TGEs infected in the GC and AC, respectively. Groups of four TGEs were infected in the GC with different infectious doses of HSV-1 17syn^+^ as indicated. A group of ten TGEs was infected in the AC with 5×10^6^ pfu. At 7 dpi, LAT copies and viral genomes/TGE were quantitated by qPCR, and the mean ratio of LAT copies/HSV-1 genomes was determined. Data are mean and SD values.

To further characterize the replication of HSV-1 in TGEs, we determined viral genome and transcript levels in infected TGEs by quantitative real-time PCR (qPCR). Infection of the GC with HSV-1 17syn^+^ resulted in the onset of a productive infection of the TGEs. As compared to 1 hpi (input genomes), the number of replicated genomes increased exponentially between 12 and 48 hpi (Figure 3A).

The viral genome and transcript levels in axonally infected TGEs were analyzed in groups of ten cultures, containing two explants each. At 6, 24, and 168 hpi with 5×10^6^ pfu of HSV-1 17syn^+^ added to the AC, the median HSV-1 DNA levels in the explants were approximately 14,000, 17,000, and 21,000 genomes/TGE, respectively (Figure 3B). Although the HSV-1 DNA load in individual TGEs varied markedly and the variations increased with time post infection, there were no significant differences in viral genome levels in the GC between 6 hpi and 168 hpi (i.e. 7 dpi).

The mode of entry into distal axons and the specificity of this qPCR-based experimental approach were tested by inhibition of the microtubule-mediated axonal transport with nocodazole and application of fusion-deficient virus particles to the AC. The addition of 10 µM nocodazole to both compartments simultaneously with viral infection of the AC led to a highly significant reduction in viral DNA levels in the explants at 2 hpi (Figure 3C). Negative effects of the nocodazol treatment on neurites within the AC were not visible by light microscopy (data not shown). Infection of the AC with 1×10^8^ particles of the gH-negative mutant HSV-1 KOSgH87 purified from the supernatants of transcomplementing gH-expressing Vero cells (Vero F6gH), corresponding to 5×10^6^ pfu on VeroF6gH cells, resulted in similar genome levels in the GC at 6 hpi to those observed in TGEs infected with HSV-1 17 syn^+^. In contrast, genomes could not be detected in TGEs after the addition of an identical number of particles of HSV-1 KOSgH87 purified from the supernatants of nontranscomplementing Vero cells (corresponding to 5 pfu on VeroF6gH cells) to the AC (Figure 3D).

Genome levels and the number of primary infected cells in TGEs were quantified in preparations of dispersed TGEs infected in the GC and the AC, respectively, in the presence of 50 µg/ml aciclovir (ACV) and harvested at 2 dpi. After infection of the AC with 1×10^6^ pfu of HSV-1 17syn^+^, approximately 2,000 HSV-1 genomes/TGE were present in the dispersed cultures. HSV-1 antigen-positive neurons or nonneuronal cells could not be detected by immunofluorescence. In contrast, infection of the GC led to the dose-dependent expression of viral antigens in neurons and nonneuronal cells (Table 1).

**Table 1:** Detection of primary infected cells in dispersed TGEs at 2 dpi.

route of infection^a^	infectious dose^b^	genomes/TGE^c^	HSV-1-positive cells/total no. of cells^d^	HSV-1-positive neurons/total no. of neurons^e^
AC	5×10^6^ pfu	1,835	-/41,000	-/445
GC	1×10^6^ pfu	20,200	392/46,000	48/500
GC	1×10^5^ pfu	6,750	34/35,000	3/549
GC	1×10^4^ pfu	3,840	1/41,000	-/589

a TGEs were infected either in the axonal or ganglion compartment (AC, GC).

b Cultures were infected with HSV-1 17syn^+^ in the presence of 50 µg/ml ACV, harvested at 2 dpi and dispersed.

c Genome levels per dispersed TGE were quantified by qPCR (mean of three experiments).

d,e HSV-1-positive cells and neurons per dispersed TGE were detected and quantified by immunofluorescence with a HSV-1-specific rabbit antiserum (d), a monoclonal antibody against the 200 kD neurofilament marker (e) and DAPI (d,e) (mean of three experiments).

Absolute and relative levels of HSV-1 IE (ICP27/UL54), early/late (E/L; gB/UL27), and late (L; gC/UL44) transcripts ranged from 500 to 125,000 copies/TGE and 1.8×10^−6^ to 6×10^−4^ (viral transcripts/ß-actin transcripts), respectively, at 6, 24 and 168 hpi (Figure 3E). IE, E/L, and L transcript levels close to the detection limit of reverse transcription (RT)-PCR were detectable in few cultures at 6 hpi, while thereafter there were moderate but significant increases in lytic transcript levels. Lytic transcripts were present in most cultures at 24 hpi and in approximately half of the cultures at 168 hpi. However, the onset of lytic gene expression in axonally-infected TGEs was remarkably low relative to the high genome levels present in the GC.

In contrast to lytic transcripts, high levels of the 2.0-kb major LAT transcript were present in all TGEs selectively infected via distal axons at 24 and 168 hpi. Between 24 and 168 hpi, LAT levels increased by approximately 40-fold. The median level of LAT at 168 hpi was approximately 1,000-fold higher than that in ICP27 transcripts (Figure 3E). LAT was also expressed in cultures infected in the GC with 5×10^6^ to 8×10^3^ pfu of HSV-1 17syn^+^. As compared to TGEs infected in the AC, the specific transcriptional activity of the LAT gene (transcripts/genome) was 350 to 8,000 -fold lower in TGEs infected in the GC (Figure 3F).

To exclude strain- and type-specific effects on HSV growth in TGEs after axonal infection, we repeated the experiments with clinical isolates of HSV-1 and HSV-2. Again, a synchronous productive infection with exponentially rising genome levels between 1 and 3 dpi was not induced in TGEs infected in the AC (data not shown).

### Stimulation of IE gene expression in axonally-infected neurons with HMBA

**Figure 4:**
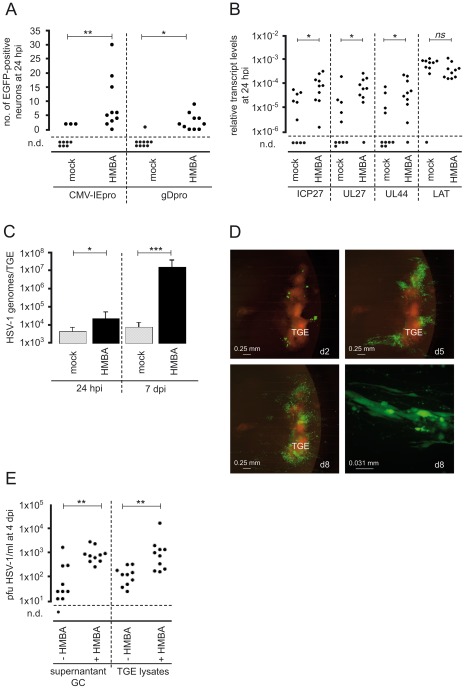
Stimulation of productive HSV-1 infection by HMBA. (A) EGFP expression in neurons in the AC at 24 hpi with 5×10^6^ pfu of HSV-1 17 CMV-IEproEGFP and HSV-1 17 gDproEGFP in the presence of 2.5 mM HMBA. Mock-treated cultures are shown as controls. n.d., No EGFP-positive neurons detected. The statistical significances of variations in the median number of EGFP-positive neurons in HMBA-treated cultures relative to mock-treated cultures are indicated (HSV-1 17 CMV-IEproEGFP, *P* = 0.0017; HSV-1 17 gDproEGFP, *P* = 0.0132; Mann-Whitney test). (B) Effect of HMBA on HSV-1 transcript levels in axonally-infected TGEs at 24 hpi. TGEs were infected in the AC with 5×10^6^ pfu of HSV-1 17 gDproEGFP. The statistical significances of differences in median transcript levels are indicated (ICP27, *P* = 0.0089; UL27, *P* = 0.0185; UL44, *P* = 0.0355; LAT, *P* = 0.1051; Mann-Whitney test). (C) Effect of HMBA on HSV-1 genome levels in axonally-infected TGEs. Groups of ten TGEs were infected in the AC with 5×10^6^ pfu of HSV-1 17 gDproEGFP in the presence of 2.5 mM HMBA; mock-treated cultures served as controls. Genome levels were determined by qPCR at 24 hpi and 7 dpi. The statistical significances of differences in median genome levels are indicated (24 hpi, *P* = 0.0115; 7 dpi, *P* = 0.0002; Mann-Whitney test). Data are mean and SD values. (D) Spread of HSV-1 in axonally-infected TGEs in the presence of HMBA. TGEs were infected in the AC with 5×10^6^ pfu of HSV-1 17 gDproEGFP in the presence of 2.5 mM HMBA and monitored daily for EGFP expression. The pattern of fluorescence in a culture with massive viral spread is shown at 2, 5, and 8 dpi (d2, d5, and d8, respectively). The bottom-right image depicts a group of HSV-1 infected nonneuronal cells at 8 dpi. (E) Effect of HMBA on the release of cell free virus. TGEs were infected in the GC with 1×10^4^ pfu of HSV-1 17 gDproEGFP in the presence or absence of 2.5 mM HMBA as indicated in culture medium without CMC and HSV-1 antiserum. At 4 dpi, supernatants (supernatant GC) and TGEs were harvested and virus was titrated. Infectious virus was liberated from TGEs by repeated freeze-thawing in 100 µl PBS (TGE lysates).

Since entry of HSV-1 into the distal axons of sensory neurons led to a predominantly nonproductive infection, we tested whether productive infection could be induced by treatment with HMBA, a known stimulator of HSV-1 IE gene expression [45]. ^T^he addition of 2.5 mM HMBA to the culture medium of axonally-infected TGEs significantly increased the number of neurons expressing EGFP under control of the CMV IE promoter and the HSV-1 gD promoter at 24 hpi (Figure 4A). Quantification of HSV-1 genome and transcript levels indicated that HMBA treatment induced viral genome replication in axonally-infected TGEs and significantly increased the expression of lytic genes at 24 hpi but did not affect the expression of LAT (Figure 4B, C). At 7 dpi, the median viral genome levels in HMBA-treated cultures were approximately 1,000-fold higher than in controls (Figure 4C). Monitoring of intact cultures demonstrated that HMBA strongly induced productive HSV-1 infection in non-neuronal cells. At 8 dpi, approximately 40% of the explants showed signs of massive viral spread (Figure 4D). When HMBA was added at 7 dpi to TGEs axonally infected with HSV-1 17 CMV-IEproEGFP, no effect on the number of EGFP-expressing cells was observed (data not shown). In order to analyze the effect of HMBA on the release of cell free infectious virus into the culture supernatants, we infected TGEs with 1×10^4^ pfu HSV-1 17 gDproEGFP in the GC using culture media without CMC and HSV-1 antiserum. As compared to nontreated explants, HMBA-treatment led to an approx. 30-fold increase in the median levels of cell free infectious virus in the supernatants at 4 dpi. The number of infectious particles released by repeated freeze-thawing of explants was approximately 10-fold higher in HMBA-treated cultures (Figure 4E).

### Transactivation of HSV-1 in axonally-infected neurons by co-infection of the GC with HSV-1 helper virus

We also tested whether genome replication and the expression of lytic genes in axonally-infected neurons could be induced by simultaneous co-infection of the GC with a helper virus. To this end we established a method for quantifying HSV-1 helper-virus-induced transcriptional transactivation and genome replication after the selective entry of HSV-1 reporter virus via distal axons. Cultures were infected in the AC with replication-competent EGFP-expressing HSV-1 mutants, whereas the GC was incubated with the spread-deficient gH-negative mutant HSV-1 KOS gH87. The use of a spread-deficient, gH-negative HSV-1 helper virus limited transactivation to those cells primarily infected by the helper virus and allowed to us to specifically follow up the replication and reporter gene expression of HSV-1 genomes present in sensory neurons after entry within the AC.

**Figure 5:**
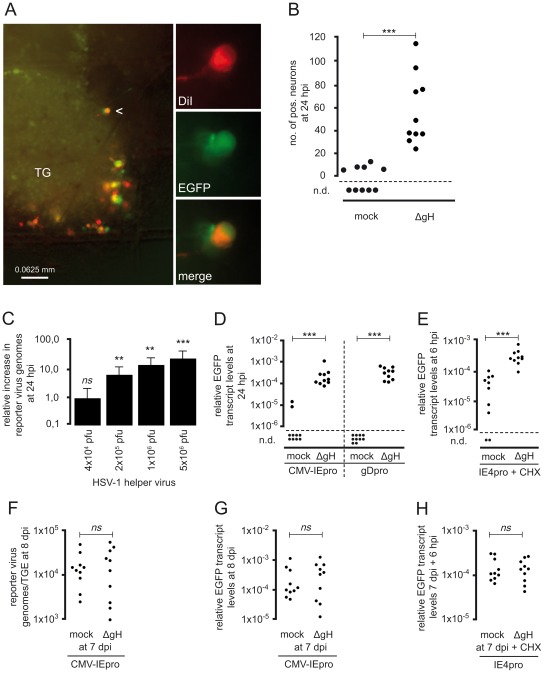
Transactivation of HSV-1 in axonally-infected TGEs by infection of the GC with HSV-1 helper virus. (A) Effect of helper virus on EGFP expression in TGEs infected in the AC with HSV-1 17 CMV-IEproEGFP. TGEs were co-infected with 1×10^6^ pfu of the gH-negative, spread-deficient mutant HSV-1 KOS gH87 in the GC and 5×10^6^ pfu of HSV-1 17 CMV-IEproEGFP in the AC. At 1 hpi, cultures were stained with DiI in the AC. The arrowhead indicates a typical DiI and EGFP double-positive neuron depicted at higher magnification in the right-hand images. (B) Effect of helper virus on EGFP expression in axonally-infected neurons. The numbers of positive neurons/culture infected in the AC with 5×10^6^ pfu of HSV-1 17 CMVpro-IE EGFP and co-infected in the GC with 5×10^6^ pfu of HSV-1 KOS gH87 (ΔgH) or mock-infected are given. Differences were highly significant (*P* = 0.0003, unpaired *t* test with Welch correction). (C) Effect of helper virus on genome replication of HSV-1 after infection of the AC. Groups of ten cultures were infected in the AC with 5×10^6^ pfu of HSV-1 17 CMV-IEproEGFP. The GC was co-infected with varying amounts of HSV-1 KOS gH87, as indicated. TGEs were harvested at 24 hpi with the helper virus, reporter virus genome levels were quantified by qPCR, and the increase of reporter virus genome levels relative to controls was calculated. The significances of helper-virus-induced increases in the median genome level of reporter virus genomes are indicated (5×10^6^ pfu helper virus, *P*<0.0001; 1×10^6^ pfu helper virus, *P* = 0.0021; 2×10^5^ pfu helper virus, *P* = 0.0015; 4×10^4^ pfu helper virus, *P* = 0.4359; Mann-Whitney test). Data are mean and SD values. (D) Reporter gene expression 24 h after co-infection of the GC with HSV-1 helper virus. Cultures were infected with 5×10^6^ pfu of HSV-1 17 CMV-IEproEGFP, and HSV-1 17 gDproEGFP in the AC. Groups of ten cultures were co-infected with 5×10^6^ pfu of HSV-1 KOS gH87 in the GC; cultures not infected with helper virus served as controls. The significances of differences in the relative transcript levels are indicated (HSV-1 CMV-IEpro EGFP, *P*<0.0001; HSV-1 gDproEGFP, *P*<0.0001; unpaired *t* test with Welch correction). (E) Helper-virus-induced transcriptional transactivation of IE gene expression in the absence of *de novo* protein synthesis. Cultures were infected in the AC with 5×10^6^ pfu of HSV-1 17 IE4proEGFP in the presence of 50 µg/ml CHX. Groups of ten cultures were co-infected in the GC with 5×10^6^ pfu of HSV-1 KOS gH87; cultures without helper-virus co-infection served as controls. TGEs were harvested in the AC at 6 hpi and the relative transcript levels of EGFP were determined. The statistical significances of differences are indicated (*P* = 0.0003; unpaired *t* test with Welch correction). (F–H) Effect of HSV helper virus added to the GC of the AC at 7 dpi. (F, G) Cultures were infected in the AC with 5×10^6^ pfu of HSV-1 17 CMV-IEproEGFP. At 7 dpi, cultures were infected in the GC with 5×10^6^ pfu of HSV-1 KOS gH87, cultures without helper-virus infection served as controls. At 24 h after the addition of helper virus, TGEs were harvested and genome levels of the reporter virus (F) and EGFP transcript levels were determined. There were no significant differences in reporter virus genome and transcript levels (genomes, *P*>0.9999, Mann-Whitney test; transcript levels, *P* = 0.7609, unpaired *t* test with Welch correction). (H) Cultures were infected in the AC with 5×10^6^ pfu of HSV-1 17 IE4proEGFP. At 7 dpi, cultures in the AC were either infected in the GC with 5×10^6^ pfu of HSV-1 KOS gH87 or mock-infected in the presence of CHX, and EGFP transcript levels were determined 6 h after addition of the helper virus. There were no significant differences in transcript levels (*P* = 0.8269, unpaired *t* test with Welch correction).

In contrast to infection of the AC with 5×10^6^ pfu of HSV-1 17 CMV-IEproEGFP only (see above), EGFP expression in TGEs was reliably induced by simultaneous co-infection of the GC with 5×10^6^ pfu of the helper virus HSV-1 KOS gH87. Staining of the AC with DiI immediately after infection with HSV-1 demonstrated that at 24 hpi, most EGFP-expressing cells exhibited a typical neuronal morphology and were stained by DiI (Figure 5A), indicating the occurrence of transactivation of silent HSV-1 infection in neurons. The number of EGFP-expressing cells was approximately 30-fold higher in TGEs co-infected with HSV-1 helper virus (Figure 5B).

EGFP DNA and RNA levels were determined by qPCR to quantify the transactivation of the reporter viruses by the HSV-1 helper virus. The simultaneous co-infection of the GC with 5×10^6^ pfu of HSV-1 KOS gH87 led to an approximately 20-fold increase in the genome levels of HSV-1 17 CMV-IEproEGFP in axonally-infected TGEs at 24 hpi (Figure 5C). Serial fivefold dilution of the helper virus revealed that a significant induction of reporter virus replication was still achieved by adding 2×10^5^ pfu helper virus to the GC.

To estimate the ratio of helper to reporter virus needed for transactivation, helper virus genome levels resulting from co-infection of the GC were quantified. Approximately 40,000 HSV-1 genomes/TGE were detected by qPCR in cultures immediately harvested after infection of the GC with 2×10^5^ pfu of HSV-1 KOS gH87 helper virus (i.e., the lowest amount of helper virus able to significantly transactivate reporter virus; see above). Comparable to what was observed with HSV-1 17syn^+^, infection of the AC with 5×10^6^ pfu of HSV-1 17 CMV-IEproEGFP reporter virus resulted in mean levels of approximately 10,000 HSV-1 genomes/TGE at 6 hpi. This corresponds to an overall ratio of helper to reporter virus genomes in co-infected TGEs of approximately 4. Considering that the HSV-1 added to the GC also infects nonneuronal cells (Figure 2A), that only a subfraction of axonally-infected neurons may be directly accessible to the spread-deficient helper virus in *en*-*bloc*-cultivated TGEs, and that the genome/pfu ratio of the helper virus preparation used was approximately 30, the actual ratio of helper to reporter virus needed to transactivate HSV-1 in axonally-infected neurons is probably much lower.

Quantification of transcript levels showed that simultaneous co-infection of the GC with HSV-1 helper virus strongly increased EGFP transcript levels at 24 hpi with HSV-1 17 CMV-IEproEGFP and HSV-1 17 gDproEGFP (Figure 5D). As observed for the induction of genome replication, at least 2×10^5^ pfu of helper virus needed to be applied to the GC to achieve a significant transactivation of EGFP expression in cultures axonally-infected with HSV-1 17 CMV-IEproEGFP (data not shown).

In order to further elucidate the mechanism underlying helper-virus-induced HSV-1 IE gene transactivation in axonally-infected TGEs, reporter gene expression in HSV-1-IE4proEGFP-infected cultures was determined in the absence of *de novo* protein synthesis at 6 hpi. As shown in Figure 5E, low levels of EGFP expression were detected in most of the cycloheximide (CHX)-treated cultures without helper virus. The simultaneous addition of 5×10^6^ pfu of helper virus significantly increased the EGFP expression, indicating that transactivation of reporter gene expression under the control of the HSV-1 ICP4 promoter occurred directly (i.e., by incoming helper virus particles and in the absence of *de novo* protein synthesis).

We also investigated whether the addition of HSV-1 helper virus to axonally-infected cultures was able to transactivate reporter virus genomes at 7 dpi (i.e., after the establishment of an LAT-positive, silent infection). These experiments showed that the HSV-1 reporter virus was refractory to transactivation by HSV-1 helper virus at 7 dpi. Infection of the GC with HSV-1 KOSgH87 did not significantly increase reporter virus genomes or gene expression in explants axonally-infected with HSV-1 17 CMV-IEproEGFP (Figure 5F, G), or directly transactivate IE gene expression in HSV-1-17-IE4proEGFP-infected cultures in the presence of CHX (Figure 5H).

### Role of VP16 in the induction of IE gene expression by HSV helper virus

**Figure 6:**
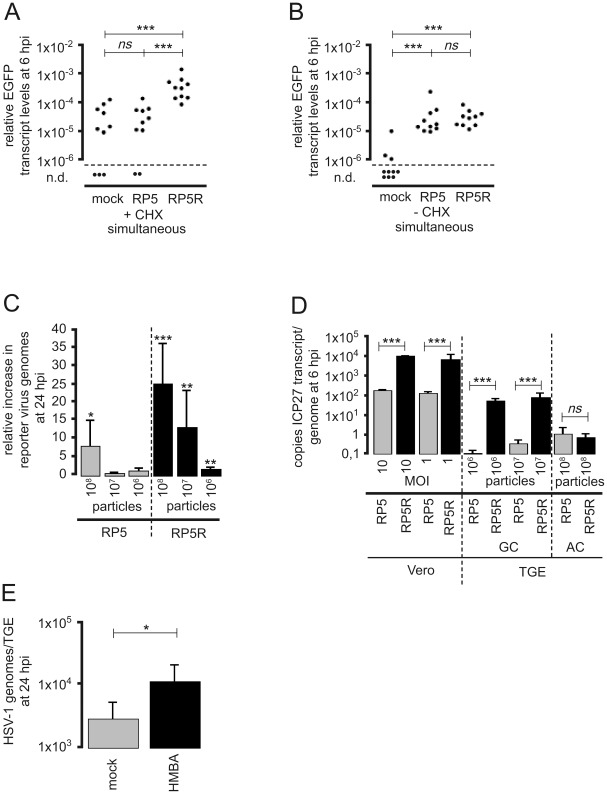
Infection of TGEs by the VP16AD-deficient mutant HSV-1 KOS RP5. (A) Transactivation of HSV-1 IE gene expression by HSV-1 KOS RP5 in the absence of *de novo* protein synthesis. Cultures were infected in the AC with 5×10^6^ pfu of HSV-1 17 IE4proEGFP in the presence of 50 µg/ml CHX. Groups of ten cultures were co-infected in the GC with identical numbers of particles of HSV-1 KOS RP5 or the revertant HSV-1 KOS RP5R (1×10^8^ particles, corresponding to 5×10^6^ pfu of HSV-1 KOS RP5R). Cultures without helper-virus co-infection served as controls. Cultures were harvested at 6 hpi, and the relative transcript levels of EGFP were determined. The statistical significances of differences in the mean relative transcript levels are indicated (mock vs. HSV-1 KOS RP5, *P* = 0.8352; mock vs. HSV-1 KOS RP5R, *P*<0.0001; HSV-1 KOS RP5 vs. HSV-1 KOS RP5R *P*<0.0001; unpaired *t* test with Welch correction), *ns* (not significant). (B) Transactivation of HSV-1 IE gene expression by HSV-1 KOS RP5 without inhibition of *de novo* protein synthesis. Cultures were infected in the absence of CHX with HSV-1 KOS RP5 and RP5R as given above (Figure 6A) and the relative transcript levels of EGFP were determined at 6 hpi. The statistical significances of differences in the mean relative transcript levels are indicated (mock vs. HSV-1 KOS RP5, *P*<0.0001; mock vs. HSV-1 KOS RP5R, *P*<0.0001; HSV-1 KOS RP5 vs. HSV-1 KOS RP5R *P* = 0.6874; unpaired *t* test with Welch correction), *ns* (not significant). (C) Transactivation of HSV-1 genome replication by HSV-1 KOS RP5. Groups of ten cultures were infected in the AC with 5×10^6^ pfu of HSV-1 17 gDproEGFP. The GC was co-infected with different infectious doses of HSV-1 KOS RP5 and RP5R as indicated. TGEs were harvested at 24 hpi with the helper virus, reporter virus genome levels were quantified by qPCR, and the increase of reporter virus genome levels relative to controls was calculated. The significances of helper-virus-induced increases in the mean genome level of reporter virus genomes are indicated (HSV-1 KOS RP5: 1×10^8^ particles, *P* = 0.0108; 1×10^7^ particles, *P* = 0.0572; 1×10^6^ particles, *P* = 0.1592; HSV-1 KOS RP5R: 1×10^8^ particles, *P*<0.0001; 1×10^7^ particles, *P* = 0.0038; 1×10^6^ particles, *P* = 0.0026; unpaired t test with Welch correction). Data are mean and SD values. (D) Specific transcriptional activity of the ICP27 gene in cultures infected with HSV-1 KOS RP5 and RP5R in the absence of *de novo* protein synthesis. Vero cells (three independent experiments) were infected with an identical number of particles of HSV-1 KOS RP5 and RP5R at the MOI indicated, groups of 4 and 10 CHX-treated TGEs were infected in the GC and AC, respectively, with the number of particles indicated, and harvested at 6 hpi. Copy numbers of the ICP27 transcript and viral genomes/TGE were quantitated by qPCR, and the ratio of ICP27 transcript copies/HSV-1 genomes was determined. The significance of differences in the specific transcriptional activity of ICP27 are indicated (Vero cells: HSV-1 KOS RP5 vs. RP5R infected at a MOI 10 and 1, *P* = 0.0001; TGEs infected in the GC: HSV-1 KOS RP5 vs. RP5R infected with 10^6^ and 10^7^ particles, *P* = 0.0001; TGEs infected in the AC: HSV-1 KOS RP5 vs. RP5R, *P* = 0.3242; unpaired t test with Welch correction). Data are mean and SD values, *ns* (not significant). (E) Effect of HMBA-treatment on genome levels in TGEs infected in the AC with HSV-1 KOS RP5. Cultures were infected in the AC with 1×10^8^ particles of HSV-1 KOS RP5 in the absence or presence of 2.5 mM HMBA. Genome levels were determined at 24 hpi. The statistical significances of differences in the mean genome level is indicated (*P* = 0.0363; unpaired *t* test with Welch correction). Data are mean and SD values.

The transactivation of the ICP4 promoter in axonally-infected and CHX-treated cultures by simultaneous infection of the GC with HSV-1 helper virus suggested a direct VP16-dependent transcriptional activation of IE gene expression. To study the role of VP16, we repeated the experiments using the VP16AD-negative mutant HSV-1 KOS RP5 as a helper virus to infect the GC. The corresponding revertant HSV-1 KOS RP5R served as a control [46]. The number of particles of HSV-1 KOS RP5 used to infect the GC was adjusted to the number of particles present in 5×10^6^ pfu of the HSV-1 KOS RP5R preparation. Following axonal infection of TGEs with HSV-1 IE4proEGFP in the presence of CHX, IE gene expression was significantly increased by infection of the GC with HSV-1 KOS RP5R, whereas the addition of RP5 did not lead to a significant transcriptional transactivation of the reporter gene at 6 hpi (Figure 6A). When added to the GC in the absence of CHX at a concentration of 10^8^ particles, both HSV-1 KOS RP5 and RP5R were able to significantly transactivate IE gene expression of the reporter virus at 6 hpi (Figure 6B). In agreement with this finding, a significant increase of reporter virus genomes was observed at 24 hpi after the addition of 10^8^ particles of HSV-1 KOS RP5 and RP5R helper viruses. Serial ten-fold dilution of the helper viruses demonstrated that the efficiency of transactivation by the rescue mutant HSV-1 KOS RP5R was 10 to 100-fold higher as compared to RP5 (Figure 6C).

The ability of HSV-1 KOS RP5 to infect distal axons was also analyzed. Similar to what was observed in TGEs at 24 hpi with HSV-1 17syn^+^, only basal levels of lytic transcripts were present in TGEs axonally-infected with HSV-1 KOS RP5, whereas LAT was expressed at high levels (data not shown). In CHX-treated, axonally infected TGEs we could not detect significant VP16-dependent differences in the transcriptional activity of ICP27 between HSV-1 KOS RP5 and RP5R at 6 hpi (Figure 6D). In contrast, the transcriptional activity of ICP27 in CHX-treated TGEs infected in the GC was approximately two log-orders higher in HSV-1 KOS RP5R-infected cultures as compared to HSV-1 KOS RP5 (Figure 6D). Similar differences between HSV-1 KOS RP5 and RP5R were observed in CHX-treated Vero cell monolayers at 6 hpi (Figure 6D). As observed with HSV-1 17syn^+^, HMBA-treatment led to a significant increase in genome levels in TGEs axonally infected with HSV-1 KOS RP5 at 24 hpi (Figure 6E).

### Transactivation of HSV-1 by co-infection of the GC with HSV-2 and PrV helper virus

**Figure 7:**
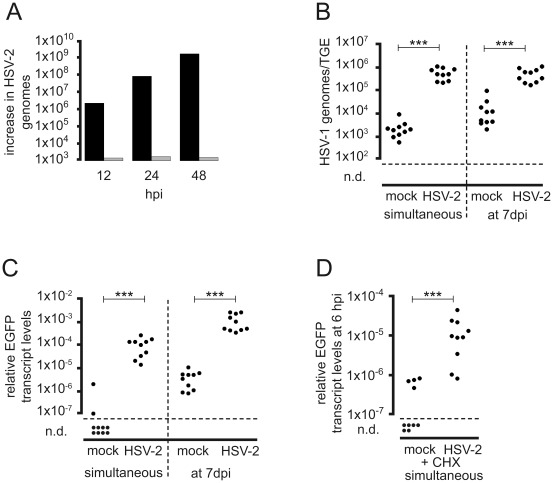
Transcriptional transactivation of HSV-1 IE gene expression by HSV-2 helper virus. (A) Replication of HSV-2 in TGEs. Groups of three TGEs were either infected in the AC with 5×10^6^ pfu of HSV-2 333 (grey bars) or in the GC with 10^4^ pfu of HSV-2 333 (black bars), and harvested at the time points indicated. DNA extracts were pooled, genome levels were determined by qPCR, and the increase in genome levels relative to 1 hpi was calculated. (B, C) Transactivation of HSV-1 by HSV-2 infection of the GC. TGEs were infected in the AC with 5×10^6^ pfu of HSV-1 CMV-IEproEGFP, and infected either simultaneously or at 7 dpi in the GC with 5×10^6^ pfu of HSV-2 333. Cultures were harvested at 24 hpi after the addition of HSV-2 to the GC, and HSV-1 genome (B) and relative EGFP transcript levels (C) were determined. Cultures not infected with helper virus served as controls. The statistical significances of differences in the transcript levels are indicated (simultaneous infection, *P*<0.0001; addition of PrV at 7 dpi, *P*<0.0001; Mann-Whitney test). (D) Transcriptional transactivation of HSV-1 by co-infection of the GC with HSV-2 in the presence of CHX. TGEs were infected in the AC with 5×10^6^ pfu of HSV-1 17 IE4proEGFP in the presence of CHX and either co-infected in the GC with 5×10^6^ pfu of HSV-2 333 or mock-treated. Cultures were harvested at 6 hpi and EGFP transcript levels normalized to β-actin transcripts were determined. The statistical significances of differences in the mean relative transcript levels are indicated (*P*>0.0001, unpaired t-test with Welch correction).

The HSV-1-independent transactivation of HSV-1 IE gene expression by co-infection of the GC with the alphaherpesviruses HSV-2 and PrV was investigated using the HSV-2 strain 333 and the replication-competent PrV mutant PrV-KaΔgGgfp. As observed with HSV-1, direct addition of HSV-2 to the GC resulted in exponentially rising genome levels indicative of massive viral replication in the TGEs, whereas addition of HSV-2 to the AC did not induce productive infection of the TGEs between 12 hpi and 48 hpi (Figure 7A). The simultaneous addition of 5×10^6^ pfu of HSV-2 helper virus to the GC of TGEs axonally-infected with HSV-1 17 CMV-IEproEGFP led to highly significant increases in HSV-1 genomes and EGFP transcript levels at 24 hpi (Figure 7 B, C). In contrast to the HSV-1 helper virus, infection of the GC with the HSV-2 helper virus at 7 dpi of the AC with HSV-1 17 CMV-IEproEGFP also strongly increased HSV-1 genomes and EGFP transcripts (Figure 7B, C). In the absence of *de novo* protein synthesis, HSV-2 helper virus strongly transactivated EGFP expression under control of the HSV-1 ICP4 promoter (Figure 7 D).

**Figure 8:**
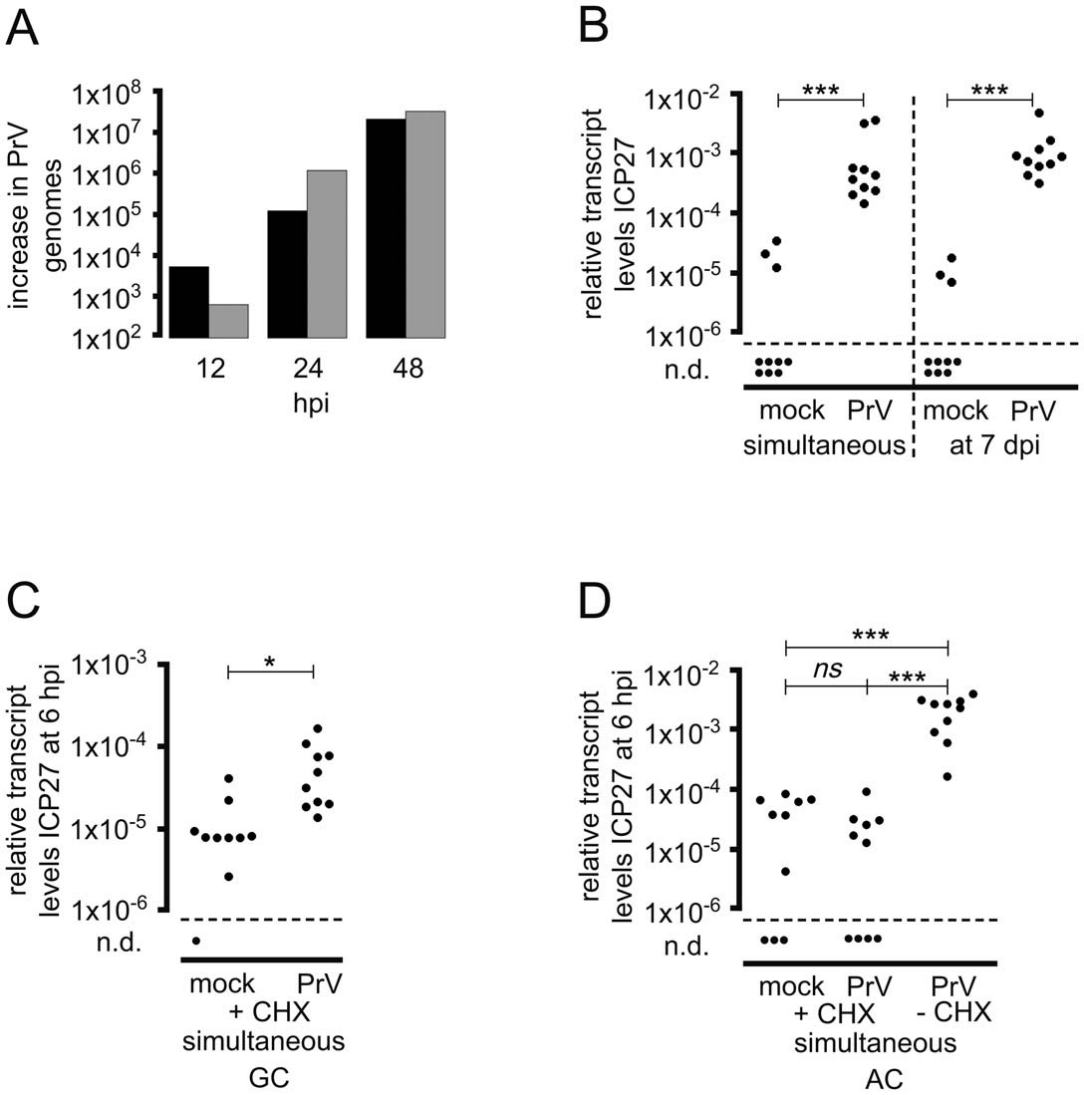
Transcriptional transactivation of HSV-1 IE gene expression by PrV helper virus. (A) Replication of PrV in TGEs. Groups of three TGEs were either infected in the AC with 5×10^6^ pfu of PrV-KaDgGgfp (grey bars) or in the GC with 10^4^ pfu of PrV-KaDgGgfp (black bars), and harvested at the time points indicated. DNA extracts were pooled, genome levels were determined by qPCR, and the increase in genome levels relative to 1 hpi was calculated. (B) Transcriptional transactivation of HSV-1 by PrV infection of the GC. TGEs were infected in the AC with 5×10^6^ pfu of HSV-1 CMV-IEproEGFP, and infected either simultaneously or at 7 dpi in the GC with 5×10^6^ pfu of PrV-KaDgGgfp. Replication of PrV was suppressed by the addition of 50 µg/ml ACV to the media. Cultures were harvested at 6 hpi after the addition of PrV to the GC, and relative ICP27 transcript levels were determined. Cultures not infected with helper virus served as controls. The statistical significances of differences in the transcript levels are indicated (simultaneous infection, *P*<0.0001; addition of PrV at 7 dpi, *P*<0.0001; unpaired *t* test with Welch correction). (C) Transcriptional transactivation of HSV-1 by co-infection of the GC with PrV in the presence of CHX. TGEs were infected in the AC with 5×10^6^ pfu of HSV-1 17 CMV-IEproEGFP in the presence of CHX and either co-infected in the GC with 5×10^6^ pfu of PrV-KaΔgGgfp or mock-treated. Cultures were harvested at 6 hpi and ICP27 transcript levels normalized to β-actin transcripts were determined. The statistical significances of differences in the mean relative transcript levels are indicated (*P* = 0.0186, unpaired *t* test with Welch correction). (D) Transcriptional transactivation of HSV-1 by co-infection of the AC with PrV. TGEs were infected in the AC with 2.5×10^6^ pfu of HSV-1 17 syn^+^ in the presence or absence of CHX and co-infected in the AC with 2.5×10^6^ pfu of PrV-KaΔgGgfp or mock-treated as indicated. Cultures were harvested at 6 hpi and ICP27 transcript levels normalized to β-actin transcripts were determined. The statistical significances of differences in the mean relative transcript levels are indicated (mock+CHX vs. PrV+CHX, *P*>0.999; mock+CHX vs. PrV−CHX, *P*<0.0001; PrV+CHX vs. PrV−CHX, *P*<0.0001; Mann-Whitney test), *ns* (not significant).

TGEs were found to be highly susceptible to direct infection of the GC with PrV. Furthermore, in contrast to HSV-1 and HSV-2, PrV infection of the AC also led to a rapid, productive spread of PrV in TGEs (Figure 8A). PrV-KaΔgGgfp proved to be a potent transactivator of HSV-1 IE gene expression in infected neurons. The simultaneous addition of 5×10^6^ pfu of PrV to the GC led to a highly significant increase in HSV-1 ICP27 gene expression at 6 hpi (Figure 8B). As observed with HSV-2 and in contrast to HSV-1 helper virus, infection of the GC with PrV helper virus at 7 dpi of the AC with HSV-1 significantly increased HSV-1 IE gene expression (Figure 8B). Simultaneous co-infection of the GC with helper virus in the presence of CHX resulted in a significant increase in the basal level of HSV-1 IE gene expression (Figure 8C). Additionally, we studied transactivation by PrV helper virus in TGEs co-infected in the AC with HSV-1 and PrV. Axonal co-infection of the TGEs resulted in a strong transactivation of HSV-1 IE gene expression by PrV helper virus at 6 hpi. Transactivation was abrogated, however, by CHX-treatment of TGEs (Figure 8D). Thus, in axonally co-infected cultures transactivation of HSV-1 appears to be dependent on *de novo* protein synthesis of PrV.

## Discussion

HSV-1 latency and reactivation *in vivo* result from a complex interaction between the virus and its host. It is assumed that immediately after virus entry into the host cell, the tegument protein VP16 plays an essential role in the combinatorial regulatory circuit controlling the lytic/latent decision. If the transactivation of IE genes by the VP16-induced complex is insufficient, this may diminish lytic gene expression in the infected neuron and favor the establishment of latency [5], [47].

As originally supposed by Roizman and Sears [48], a lack of retrograde axonal transport of VP16 upon entry into the distal axons could be responsible for the poor onset of IE gene expression in neurons *in vivo*. By contrast, infection of the soma may enable VP16 to reach the nucleus and to initiate a lytic infection of the neuron, as observed in neuronal cultures non-selectively infected *in vitro*.

In addition, transactivation by residual VP16 reaching the soma of the axonally infected neuron may be blocked. Modulation of the function of VP16 by other components of the HSV-1 outer tegument layer might contribute to the insufficiency of transactivation of IE gene expression in axonally-infected neurons. The tegument proteins UL46 and UL47, which reportedly increase the activity of VP16 [49], [50], are predominantly lost from capsids during retrograde axonal transport [22]. In addition, residual VP16 activity may be blocked by the cytoplasmic sequestration of HCF-1 in unstimulated neurons [51], and the interaction of HCF-1 with the basic leucine zipper proteins, Luman and Zhangfei [52]–[54].

Various cell culture systems have been used to study the establishment of and reactivation from latency at the molecular level *in vitro* [5]. If a missing or strongly reduced axonal transport of VP16 is important for the lytic/latent decision in the infected neuron, the ability of HSV-1 to induce lytic gene expression in neurons should differ with the site of viral entry. Specifically, the selective infection of distal axons should predispose neurons to a nonproductive HSV-1 infection. To our knowledge, and contrary to this assumption, differences specific to the site of entry in the ability of HSV to cause productive infection of neurons remain unknown. The selective entry of HSV-1 into the axons of dissociated DRGs *in vitro* has been reported to result in the productive infection of neurons, and consequently in the progressive spread of HSV throughout the cultures [42], [55].

While a predominantly nonproductive HSV-1 infection can be spontaneously established in neuronal cultures [56], acute lytic infection must usually be suppressed for some time to enable the efficient establishment of latent infection *in vitro*, especially if infections are performed at a high MOI [57]–[61]. As shown by Bertke et al. [62] neuronal factors may have a significant impact on the relative permissiveness for productive infection. Thus, adult murine A5-positive sensory neurons are nonpermissive for productive infection with HSV-1 in vitro. To our knowledge, chicken trigeminal neurons have not been tested for the expression of the A5 (Galβ1-4GlcNAc-R) epitope. As additional markers of adult murine trigeminal neurons preferentially harboring latent HSV-1, the calcitonin-gene related peptide (CGRP) and the high affinity NGF receptor (Trk_A_) have been described [63]. The majority of neurons in the adult chicken trigeminal ganglion are reactive for the calcitonin-gene related peptide (CGRP) [64]. Furthermore, basic characteristics of neurotrophins and their receptors in sensory neurons appear to be well preserved between mammals and the chick, e.g., the same genes are found and expressed with shared common features in different neuron populations during development. Trk_A_ is expressed at high levels in the embryonic chicken TG [65].

Analysis of the viral transcript and genome levels in HSV-1 infected embryonic chicken TGEs between 6 hpi and 7 dpi revealed that the infection of free distal axons spontaneously resulted in a largely nonproductive HSV-1 infection that was characterized by stationary genome levels with a median of approximately 10,000 to 20,000 genomes/TGE infected with 5×10^6^ pfu HSV-1 17syn^+^, the expression of low and fluctuating levels of lytic transcripts, and the prominent expression of LAT at 24 hpi and thereafter. A general lack of susceptibility of the organ model to HSV-1 is unlikely to account for this observation. Previous studies showed that neuronal embryonic chicken tissues are permissive for HSV-1, and are a suitable model system for the analysis of neurotropic alphaherpesviruses [44], [66]. Our data confirm that HSV-1 can efficiently infect the distal axons of embryonic chicken TGEs. We estimate that the addition of HSV-1 at saturating infectious doses to the AC results in stable mean levels of 70–210 HSV-1 genomes per neuron with neurites reaching the AC. This is highly consistent with levels of approximately 70 and 180 viral genomes per latently infected LAT-negative and LAT-positive neuron in the mouse model reported by Chen et al. [67]. Fusion-incompetent viral particles added to the AC of embryonic chicken TGEs were not transported in a retrograde direction to the GC. Thus, in agreement with earlier studies, HSV-1 enters the distal axons of embryonic chicken TGEs by direct fusion with the axonal membrane, and not via endocytotic uptake [22], [68]–[70].

If this is correct, a lack of axonal transport of VP16 should result in a similar pattern of lytic gene expression in neurons axonally-infected with wt HSV and a VP16AD-negative mutant. In fact, ICP27 transcript levels in CHX-treated TGEs axonally infected with HSV-1 KOS RP5 and RP5R did not differ. Furthermore, levels of lytic transcripts at 24 hpi were found to be comparable in cultures infected with HSV-1 17syn^+^ and various EGFP-expressing HSV-1 17 mutants, HSV-1 KOS RP5 and RP5R (data not shown). Thus, at least in the early phase of infection, conspicuous VP16-dependent differences in lytic viral transcript levels were not evident in axonally-infected TGEs. Proenca et al. [71] recently demonstrated that HSV-1 IE gene expression by a VP16-independent mechanism can precede the establishment of latency in sensory neurons. In addition, we also found similar levels of LAT expressed by HSV-1 wt virus and the VP16AD-negative mutant in axonally-infected TGEs.

In the absence of functional VP16, HMBA has been described to enhance IE gene expression and lytic growth of HSV-1, especially if cells are infected at low MOI [45], [72]. The exact mode of action of the cytodifferentiating agent HMBA on HSV-1 growth is not fully understood. HMBA induces the differentiation of murine erythroleukemia cells (MELC) and other transformed cells to a less transformed phenotype [73]. Comparison of the effects of various other agents known to promote the differentiation of MELCs demonstrated that some of these substances complement the growth of VP16-deficient HSV-1 whereas others antagonize the effect of HMBA [74]. HMBA-treatment also increases the replication of VP16-positive HSV-1 and HSV-2 in epidermal and neuronal cells [75], and the oncolytic activity of a gamma1-34.5-negative HSV-1 mutant in oral squamous carcinoma cells [76].

Accordingly, HMBA treatment significantly increased the expression of lytic genes in TGEs axonally-infected with the VP16AD-negative mutant HSV-1 KOS RP5. HMBA treatment also restored the ability of VP16-competent HSV-1 strains to initiate a productive infection of sensory neurons and stimulated viral spread within nonneuronal cells. In TGEs axonally-infected with the spread-deficient mutant HSV-1 KOS gH87, HMBA treatment significantly enhanced the expression of lytic genes (data not shown). Together these findings provide evidence of insufficient transactivation of IE gene expression by VP16 in axonally-infected neurons.

With respect to the ability of HSV-1 to induce a productive infection of neurons when added directly to the GC, we reasoned that silent HSV-1 infection resulting from axonal infection could be transactivated by the direct co-infection of neurons with HSV-1 helper virus. Our results show that both genome replication of the reporter virus and reporter gene expression under the control of HSV-1 IE, E/L, and human CMV (HCMV) IE promoters can be transactivated by simultaneous infection with a helper virus introduced into the GC. Quantification of helper and reporter virus levels in co-infected TGEs indicated that only a few helper virus particles appear to be sufficient to transactivate the reporter virus in neurons.

These results further support the hypothesis that the poor expression of lytic genes after axonal entry is due to low or missing transactivation by VP16, since helper-virus-induced transactivation of IE gene expression by simultaneous co-infection of the GC occurred in the absence of de novo protein synthesis, and efficient transactivation was dependent on the presence of functional VP16 in the HSV-1 helper virus.

The related alphaherpesviruses HSV-2 and PrV also efficiently transactivated lytic HSV-1 gene expression in axonally-infected neurons. However, unlike HSV-1 and HSV-2, the lytic gene expression after axonal entry of PrV is sufficient to initiate a synchronous, productive infection of neurons. The rapid spread of PrV in the explants following axonal infection is highly consistent with its high *in vivo* pathogenicity, which is substantially greater than that of HSV [77].

The simultaneous co-infection of the GC with HSV-2 or PrV was found to directly transactivate HSV-1 IE gene expression in neurons in the absence of *de novo* protein synthesis. Transactivation of HSV-1 IE gene expression is most likely mediated by HSV-2 VP16 and the PrV VP16/pUL48 homologue [78], although it has been reported that UV-inactivated PrV particles do not transactivate the expression of reporter genes controlled by IE promoters of HSV-1 or PrV [79], [80]. Interestingly, the HSV-2 and PrV but not HSV-1 helper viruses were still able to efficiently transactivate HSV reporter virus gene expression at 7 dpi. It is unclear why HSV-1 helper virus specifically fails to transactivate HSV-1 genomes in axonally-infected neurons when added after the establishment of a silent, latency-like infection in the cultures. However, our finding is in agreement with the results of Su et al. [81], who reported that HSV-1 genomes in long-term NGF-differentiated, quiescently-infected PC12 cells become refractory to superinfection with HSV-1. Mador et al. [82] showed that the expression of HSV-1 LAT renders neurons resistant to infection with HSV-1 but not HSV-2, and concluded that protection against superinfection with HSV-1 may be an important function of HSV-1 LAT. Since axonally-infected TGEs express high levels of LAT at 7 dpi, a LAT-dependent mechanism may be responsible for the HSV-1-specific failure to transactivate silent HSV-1 infection observed in this study. As discussed by Berngruber et al. [83], protection of the host cell harboring latent virus by superinfection inhibition is not restricted to bacteriophages but appears to be a common mechanism in the maintenance of latency in many viral systems including HSV-1, and may be a driving force in the evolution of viral latency.

The use of fluorescence microscopy to monitor intact, axonally-infected TGEs with the replication-competent mutant HSV-1 17 CMV-IEproEGFP showed that a few isolated neurons expressed EGFP within the first days after infection. Monitoring the onset of EGFP expression in individual neurons revealed that this was associated with an approximately fivefold increase in the cumulative number of EGFP-positive neurons during the first 10 days after axonal infection. The cumulative number of EGFP-positive neurons increased at a small but constant rate during weeks 3–10 after axonal infection. Balliet et al. [84] interpreted the *de novo* onset of EGFP expression under the control of the HCMV IE promoter in latently HSV-1-infected neurons as spontaneous molecular reactivation, a term coined by Feldman et al. [85]. Spontaneous molecular reactivation, as observed in the mouse TG, might reflect asymptomatic viral shedding and recurrent disease in humans occurring in the absence of known external stimuli of HSV reactivation [86]. Stochastic spontaneous reactivation patterns have also been described in other latent herpesvirus infections [87]. Since HSV-1 spreads from EGFP-positive neurons to surrounding nonneuronal cells in a small proportion of axonally-infected embryonic chicken TGEs, at least a fraction of the former must have been productively infected.

Interestingly, after a short period of expansion, the growth of these plaque-like clusters of infected cells ceased and EGFP-positive cells finally disappeared again. This is reminiscent of the results of Barreca and O'Hare [88], [89], who reported that HSV-1-infected MDBK cells develop an interferon-dependent, refractory state leading to the suppression of viral growth on the one hand and the persistence of virus in a subpopulation of cells on the other.

As reported by Camarena et al. [90], continuous NGF-mediated signaling is required to maintain HSV-1 latency in primary neuron cultures. The use of serum-free culture media supplemented with NGF as the only growth factor may thus have favored the long-term nonproductive infection of neurons observed in the present study. The presence of fetal calf serum (FCS) in the culture medium and continuing viral spread from infected epithelial cells to distal axons instead may enhance the productive HSV-1 infection of embryonic chicken TGEs [43]. In the present study we found that treating the cultures with the cell-differentiation-inducing agent HMBA not only enhanced the expression of lytic genes in axonally-infected neurons but also strongly promoted secondary viral spread to the surrounding neurons and nonneuronal cells. Clearly, differentiation, trophic state, developmental stage, and age of neurons and nonneuronal cells significantly impact the outcome of HSV-1 infection in neuronal organotypic cultures. In particular, even if the vast majority of neurons become nonproductively infected, conditions favoring the massive secondary spread of HSV-1 will disrupt the establishment of a long-lasting latency-like HSV-1 infection as long as a few productively infected neurons and susceptible cells are present in the organotypic cultures. Thus, the apparent failure of HSV-1 to spontaneously establish a latency-like infection upon axonal infection in earlier studies may also reflect differences in the endogenous capability of cultures to establish a refractory antiviral state.

In conclusion, our work provides direct experimental evidence that HSV-1 entry into the distal axons of sensory neurons is characterized by a site-of-entry-specific restriction of lytic gene expression. We consider our observations to be relevant for the course of natural HSV-1 infection and the underlying mechanism to be decisive for the establishment of latency. Although the block to lytic infection is not complete, switching off the transactivation of IE genes by VP16 is probably an essential first step in the combinatory circuit that ultimately triggers the establishment of latency in sensory neurons infected via the physiological route. In turn, the repression of massive lytic replication in the PNS despite infection of neurons at a high MOI may enable the host to initiate an innate immune response and prevent anterograde spread of the virus until adaptive immune mechanisms have developed [91]–[93]. In this way, both the efficient establishment of latent infection and the prevention of excessive viral pathogenicity might be warranted.

## Materials and Methods

### Ethics statement

All procedures involving chicken embryos complied with the relevant national guidelines. Embryonated chicken eggs are not subject to the restrictions imposed by German laws related to animal protection. Furthermore, any organ explanted from a killed animal is not defined as an animal experiment (Tierschutzgesetz §7 (1)). For infection experiments, explant cultures were treated with the virus, and thus no infection of live organisms occurred. The use of chicken embryos has been approved officially as an alternative to replace animal experiments, in accordance with the replacement, refinement, and reduction strategy for animal experiments of the EU.

### Generation of EGFP-expressing recombinant HSV-1

#### Cloning of the transfer plasmids

To generate mutant HSV-1-expressing EGFP under the control of the CMV IE-promoter, the HSV-1 ICP4 promoter, and the HSV-1 gD promoter, transfer plasmids with the respective reporter cassettes inserted between the HSV-1 UL21 and UL22 (gH) genes were constructed.

The transfer plasmid pUCgHBgl2m_CIE-EGFP, which was used to generate HSV-1 17 CMV-IEproEGFP expressing EGFP under the control of the CMV IE promoter, was cloned as follows. The vector pUCgHBgl2mABS was generated by PCR-mediated, site-directed mutagenesis of pUCgHBgl2m [94] comprising the gH coding sequence within the HSV-1 BglIIm fragment (6.4 kb, from nucleotide positions 41,449–47,855 of the HSV-1 genome; GenBank accession no. X14112) [95]. pUCgHBgl2mABS contains a unique BclI site immediately downstream of the gH stop codon (nucleotide position 43,866), a second poly A region derived from plasmid pEGFP-1 (BD Biosciences Clontech, Heidelberg, Germany), and a unique SpeI site 5′ of the endogenous gH poly A region. The transfer plasmid pUCgHBgl2m_CIE-EGFP was obtained by subcloning the 0.79-kb HindIII/XbaI EGFP-coding fragment of pEGFP-1 into the HindIII/XbaI sites of pVAX1 (BD Biosciences Clontech) and transferring the 1.45-kb SpeI/XbaI fragment containing the HCMV major IE promoter region of pVAX1 and the EGFP coding sequences into pUCgHBgl2mABS.

The transfer plasmid pUCgHBgl2m_IE4-EGFP was constructed to generate HSV-1 17 IE4proEGFP expressing EGFP under the control of the HSV-1 IE ICP4 promoter. A PCR product containing a 380-bp fragment of the promoter and the 5′-nontranslated region of ICP4 (positions 131,079–131,458; nucleotides −350 to +30 relative to the transcript start site) [96] was inserted into pEGFP-1, and the 1.14-kb Spe1/Xba1 ICP4 promoter/EGFP fragment was subcloned into pUCgHBgl2mABS.

The transfer plasmid pUCgHBgl2m_gDproEGFP was constructed to generate HSV-1 17 gDproEGFP expressing EGFP under the control of the HSV-1 E/L gD promoter. A PCR product containing a 461-bp fragment of the promoter and 5′-nontranslated region of gD (positions 137,941–138,402; nucleotides −392 to +69 relative to the transcript start site) [95] was inserted into pEGFP-1, and the 1.22-kb Spe1/Xba1 gD promoter/EGFP fragment was subcloned into pUCgHBgl2mABS.

#### Homologous recombination and selection of the recombinant virus

Recombinant HSV-1 expressing EGFP was generated by cotransfecting Vero cells with purified DNA of the HSV-1 strain 17syn^+^ and plasmids pUCgHBgl2m_CIE-EGFP, pUCgHBgl2m_IE4pro-EGFP, and pUCgHBgl2m_gDproEGFP. For each construct, several clones of the recombinant viruses (e.g., HSV-1 17 CMV-IEpro-EGFP, HSV-1 17 IE4pro-EGFP, and HSV-1 17 gDpro-EGFP) exhibiting bright autofluorescence were selected and plaque-purified to homogeneity. The correctness of the insertion of the reporter protein cassette into the viral genome was verified by restriction enzyme analysis and DNA sequencing (data not shown).

### Cells, viruses, and chemicals

TGEs and standard cell lines were cultivated at 37°C in an atmosphere of 5% CO_2_ and 100% humidity. Vero and RK13 cells were grown in minimal essential medium (MEM; Biochrom, Berlin, Germany) supplemented with 10% v/v FCS and antibiotic/antimycotic additives (100 µg/ml streptomycin, 100 U/ml penicillin G, and 0.25 µg/ml amphotericin B; Invitrogen, Karlsruhe, Germany). TGEs were grown in TG medium consisting of d-valine-modified MEM (Biochrom) with 5 g/l glucose, 200 mM l-glutamine (Sigma-Aldrich, Munich, Germany), 50 ng/ml rat NGF 7S (Invitrogen), and antibiotic/antimycotic additives. The carboxymethylcellulose (CMC)-containing TG medium comprised MEM with 0.5% CMC (Sigma-Aldrich), 5 g/l glucose (Sigma-Aldrich), 50 ng/ml NGF 7S, 1% human serum (Harlan Sera-Lab, Loughborough, UK), and antibiotic/antimycotic additives.

The HSV-1 strain 17syn^+^, EGFP-expressing HSV-1 recombinants and HSV-2 333 were propagated on Vero cells, and infection titers were quantified using a plaque assay.

The gH-negative mutant HSV-1 KOS gH87 in which the 1,790-bp NcoI-XbaI-fragment of the gH gene has been replaced by the lacZ gene under control of the CMV IE promoter was kindly provided by P.G. Spear (Chicago, IL, USA). Fusion-competent stocks of HSV-1 KOS gH87 were propagated and titrated on gH-expressing VeroF6gH cells kindly provided by A.C. Minson (Cambridge, UK) [97]. Fusion-deficient virus particles were collected from the supernatants of nontranscomplementing Vero cells.

Enrichment and purification of HSV-1 particles from cell-culture supernatants by ultracentrifugation on a 20% saccharose cushion was performed as described previously [94].

The VP16AD-negative strain HSV-1 KOS RP5 and the corresponding revertant RP5R were generated and propagated as described by Tal-Singer et al. [46], and kindly provided by S.J. Triezenberg, Van Andel Research Institute, Grand Rapids, MI, USA. For infection of TGEs, HSV-1 KOS RP5 and RP5R were grown and titrated on Vero cells.

Using 204-nm latex polystyrene beads (Plano, Wetzlar, Germany) as a calibration standard, the particle/pfu ratios of virus stocks of HSV-1 KOS RP5, HSV-1 KOS RP5R, and HSV-1 17syn^+^ propagated on Vero cells were determined to be 10,000, 31.5, and 18, respectively. The particle/pfu ratios of purified particles of HSV-1 KOS gH87 propagated on VeroF6gH and Vero cells were 15.5 and 2×10^7^, respectively.

Generation of the EGFP-expressing mutant PrV-KaΔgGgfp is described elsewhere [98], and the virus was propagated on RK13 (rabbit kidney) cells. CHX, nocodazole, acyclovir, HMBA, and other chemicals used for cell culture were purchased from Sigma-Aldrich.

Clinical isolates of HSV-1 and HSV-2 were obtained from the routine diagnostic laboratory of the University Hospital of Münster. Isolates were propagated on Vero cells as described above.

### TG explantation and culture

Day-15 chick embryos were used for the preparation of TGs. The skull was opened dorsally, the brain removed, and the TG dissected out and maintained in MEM at 4°C until being used further. Tissue culture dishes (35 mm in diameter) were coated with 10 mg/ml gelatin (Biochrom) followed by 20 µg/cm^2^ mouse laminin (Roche, Mannheim, Germany). They were then washed and dried. The bottom of the coated tissue-culture dishes was scratched with a pin rake (Tyler Research, Edmonton, Canada) to form equally placed grooves. A cloning cylinder (Omnilab, Bremen, Germany) that was 8 mm high, and with an internal diameter of 8 mm and a wall thickness of 1 mm, was attached to the center of the culture dish with sterile silicone grease (Bayer, Leverkusen, Germany), forming inner and outer compartments. TGEs were fixed to the inner chamber by 1–2 min of air-drying, and then both compartments were supplied with TG medium and incubated under standard conditions for 5 days. During TGE fixation, care was taken to ensure that the pole of explants containing axons projecting to the periphery *in vivo* faced the inner rim of the diffusion barrier. Axon penetration from the inner to the outer compartment was monitored visually prior to infection.

For selective staining of neurons innervating the external compartment, 5 µl of the retrograde axonal tracer DiI (Vybrant DiI cell-labeling solution, Invitrogen) was added per milliliter of culture medium and incubated for 20 min.

### Infection of TGEs

Before the selective infection of sensory nerve endings in the AC, the medium in the GC was replaced by 0.3 ml of CMC-containing TG medium (as described above). After 30 min, the virus suspended in 2 ml of TG medium was added to the AC, where it was incubated for 1 h at 37°C. The virus inoculum was then removed, the AC washed once with phosphate-buffered saline (PBS), and CMC-containing TG medium was added. Cross-contamination by leakage from the AC was avoided by keeping the fluid level slightly higher in the GC than in the AC.

For nonselective infection of TGEs, the medium in the GC was carefully aspirated and virus suspension (0.3 ml) was added. At 1 hpi, the virus inoculum was replaced by 0.3 ml of CMC-containing TG medium.

For infection of the GC with helper virus, the medium of the inner chamber was carefully aspirated, and 0.3 ml of helper virus suspension was added. After 45 min, the helper virus inoculum was replaced by 0.3 ml of CMC-containing TG medium, in which cultures were incubated at 37°C. To avoid cross-contamination, co-infection of the AC with reporter virus was performed 20 min after completing infection of the GC with helper virus and the addition of CMC-containing TG medium to the GC. At 1 h after incubation of the AC with 2 ml of reporter virus inoculum at 37°C, the virus inoculum was aspirated from the AC, cultures were washed once with PBS, and CMC-containing TG medium was added. The chambers were incubated at 37°C for the duration of the experiments.

### qPCR

#### Nucleic acid extraction and complementary DNA synthesis

Chicken TGEs were removed en bloc from culture dishes using sterile, nucleic-acid contamination-free forceps, placed immediately in RNAlater stabilization reagent (Qiagen, Hilden, Germany) and stored according to the manufacturer's instructions until being used further. To isolate nucleic acids from en bloc tissue, cultivated TGEs were dissected and homogenized in RLT lysis buffer containing β-mercaptoethanol (AllPrep Micro Kit, Qiagen) using the TissueRuptor system (Qiagen). Genomic DNA and total RNA were subsequently purified on AllPrep DNA spin columns and RNeasy MinElute spin columns according to the manufacturer's instructions.

Complementary DNA (cDNA) was prepared from total RNA extracts with the aid of the Superscript III First-Strand Synthesis System for RT-PCR (Invitrogen), applying 50 ng per reaction of the random hexamer primers included in the kit. Samples were treated with RNase H (Invitrogen) to increase the sensitivity of subsequent PCR steps. Control reactions included samples with reverse transcriptase omitted.

#### Real-time PCR analysis

Real-time PCR amplification and detection were performed on a LightCycler 2.0 instrument (Roche) in 20-µl volumes containing 2 µl of cDNA reactions or 5 µl of DNA eluates, PCR reagents, target-specific primers, and (for HSV-1 transcripts and EGFP) dual-labeled fluorescent probes (Biomers, Ulm, Germany). Chicken β-actin PCR was carried out using the primers at 0.2 µM as reported by Philbin et al. [99], and the LightCycler FastStart DNA Master^Plus^ SYBR Green I Kit (Roche) according to the manufacturer's instructions. The specificity of the amplified products was assessed by performing melting-curve analyses. DNA sequences and transcripts of the HSV-1 genes ICP27/UL54 and UL44/gC, and the 2.0-kb intron of LAT were quantified using the primers at 0.4 µM and the dual-labeled probes at 0.2 µM, as described by Cohrs et al. [100]. UL27/gB sequences were amplified using the primers at 0.5 µM and the dual-labeled probe at 0.2 µM, as reported by Mador et al. [101]. A 75-bp region of the EGFP transgene was amplified using the primers at 0.5 µM and the dual-labeled probe at 0.1 µM, as described previously by Pan et al. [102]. Probes were labeled with the fluorescent dye 6-carboxyfluorescein at the 5′ end and with the quencher dye 6-carboxytetramethylrhodamine at the 3′ end. PCRs using hydrolysis probes were set up with the LightCycler TaqMan Master Kit (Roche) following the manufacturer's protocol. All PCR runs comprised a hot-start preincubation cycle and 45 quantification cycles.

Variations in the efficiency of nucleic acid extraction were taken into account by coamplification of the housekeeping gene β-actin, as reported by Bode et al. [103]. Samples containing conspicuously low levels of β-actin genomic DNA and/or cDNA levels (e.g., more than 3 crossing point (*C*_p_) values difference compared to average samples) were treated as outliers and excluded from further experiments. For the determination of relative levels of viral transcripts, *C*_p_ values of viral target sequences were normalized to threshold cycles of chicken β-actin 
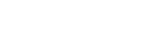
, according to Bode et al. [103]. Standard curves for the quantification of HSV-1 genes and EGFP were generated from tenfold serial dilutions of purified gDNA of HSV-1 17 syn^+^ and pEGFP-1 vector, respectively.

Data were analyzed statistically using InStat software (version 3.0b, Graph Pad, La Jolla, CA, USA). HSV-1 DNA-negative samples were excluded from statistical analyses of HSV-1 genome and transcript levels. The significance of differences in genome levels was determined by comparing the number of genomes per TGE. The significance of differences between relative levels of viral transcripts was calculated by comparing differences in *C*_p_ values (*C*_p_ β-actin−*C*_p_ transcript). *C*_p_ values of 40.01 and 41 were applied to samples containing transcript levels below the linear range of qPCR (*C*_p_>40) or with no detectable levels of viral transcripts, respectively.

In order to determine the detection limit of RT-PCR assays performed on samples from infected cells, Vero cells were infected with HSV-1 17 CMV-IEproEGFP at an MOI of 1 and serial tenfold dilutions for 1 h at 37°C. At 3 hpi, cells were trypsinized and washed with PBS. Genomic DNA and RNA were extracted from 5×10^5^ cells using the Allprep DNA/RNA Mini Kit (Qiagen), and cDNA was synthesized as described above. Both ICP27 and EGFP were amplified by qPCR using triplicate samples of gDNA and cDNA, respectively. The lower limit of the linear range of quantification was found to lie between 50 and 100 copies of gDNA per 20 µl of PCR reaction mixture, while transcripts could still be measured in samples from cells infected at an MOI of 10^−5^.

HSV-2 genomes in infected TGEs were quantitated using the Light Cycler HSV-1/2 Qual Kit (Roche) and internal quantitative standards consisting of serially tenfold diluted, purified HSV-2 333 DNA.

### Immunofluorescence

TGEs were fixed at different times post infection for 3 h in 4% phosphate-buffered paraformaldehyde (pH 7.4) at 4°C. Fixed TGEs were embedded in Tissue-Tek (Sakura Finetek, Zoeterwoude, The Netherlands) and frozen in isopentane cooled in liquid nitrogen. Cryosections were cut at a thickness of 7 µm on a cryotome (CM3050, Leica, Bensheim, Germany) and mounted on coated slides (Superfrost Plus, Langenbrinck, Emmendingen, Germany). Fluorescence immunohistochemistry was performed on frozen sections using previously described standard protocols [104]. The polyclonal anti-HSV-1 antibody (Dako, Glostrup, Denmark) was diluted to 1∶200 and incubated with the sections overnight at 4°C. A biotinylated α-rabbit secondary antibody (Vector Laboratories, Burlingame, CA, USA) was used, followed by fluorescence-signal detection with A488-streptavidin (Molecular Probes, Leiden, Netherlands). Nuclei were stained using a mounting medium containing DAPI (Vector Laboratories). Sections were examined with a fluorescence microscope (DM, Leica), and images were digitized and transferred to a computer using a Diagnostic Instruments SPOT II camera system (Visitron, Munich, Germany). The monochrome fluorescence signals were merged into a single multicolor image using SPOT II software.

Preparations of dispersed TGEs were obtained, processed for immunofluorescence and stained with HSV-1-specific rabbit antiserum, a monoclonal antibody against the 200 kD neurofilament marker (Roche) and DAPI as described by Hafezi et al. [43].

### Fluorescence imaging of intact TGEs

Daily monitoring of EGFP expression was performed with a fluorescence microscope (Axiovert 200, Zeiss, Jena, Germany) using a bandpass filter for EGFP (AHF Analysentechnik, Tübingen, Germany). Images were obtained using an AxioCam MRm camera (Zeiss) and AxioVision 4.6 software (Zeiss).
